# CNN-Based Suppression of False Contour and Color Distortion in Bit-Depth Enhancement †

**DOI:** 10.3390/s21020416

**Published:** 2021-01-08

**Authors:** Changmeng Peng, Luting Cai, Xiaoyang Huang, Zhizhong Fu, Jin Xu, Xiaofeng Li

**Affiliations:** 1 School of Information and Communication Engineering, University of Electronic Science and Technology of China (UESTC), 2006 Xiyuan Avenue, Chengdu 611731, China; pengcm@std.uestc.edu.cn (C.P.); hxy@std.uestc.edu.cn (X.H.); jinxu@uestc.edu.cn (J.X.); xfli@uestc.edu.cn (X.L.); 2 ZTE Corporation, Chongqing 401121, China; clt19951229@gmail.com

**Keywords:** bit-depth enhancement, Visual Internet of Things, convolution neural network, false contour, color distortion

## Abstract

It is a challenge to transmit and store the massive visual data generated in the Visual Internet of Things (VIoT), so the compression of the visual data is of great significance to VIoT. Compressing bit-depth of images is very cost-effective to reduce the large volume of visual data. However, compressing the bit-depth will introduce false contour, and color distortion would occur in the reconstructed image. False contour and color distortion suppression become critical issues of the bit-depth enhancement in VIoT. To solve these problems, a *Bit-depth Enhancement method with AUTO-encoder-like structure* (BE-AUTO) is proposed in this paper. Based on the convolution-combined-with-deconvolution codec and global skip of BE-AUTO, this method can effectively suppress false contour and color distortion, thus achieving the state-of-the-art objective metric and visual quality in the reconstructed images, making it more suitable for bit-depth enhancement in VIoT.

## 1. Introduction

Visual sensors can provide richer and more intuitive information compared to other types of sensors. They are critical *Perception Front End*s (PFEs) of VIoT, and have been used in many scenarios, such as security surveillance, person identification, image retrieval, and telemedicine [1,2]. However, compared with other types of signals, such as temperature sensor signals, visual signals have a huge amount of data. The surveillance system consisting of 6 million cameras has an hourly data volume of about 7.5 petabytes [3]. It is a great challenge to transmit and store these visual data.

Usually, each PFE in VIoT can only supply the compact memory space, limited energy and computing resources. Therefore, the visual signals perceived by PFE must be sent to the cloud platform with massive storage space and rich computing resources [1]. Even with the help of high-efficient compression technology, such as H.26x, in VIoT application environment, compression of massive VIoT data will occupy a lot of bandwidth and consume a lot of energy, which is a heavy burden for the green environmental system. Therefore, based on the existing video data encoding and decoding framework, it is very necessary to do proper preprocessing before the visual data is compressed [4].

Existing preprocessing methods have high complexity and are not suitable for the limited resource application environment of VIoT. The advantage of compressive sensing (CS) is that it can perform data sampling and compression at the same time, which makes it widely investigated in IoT scenarios [5,6,7,8,9]. However, resources required to calculate, store the measurement matrix and compression process are huge burdens for VIoT. With the popularity of deep learning, *Convolution Neural Network* (CNN) feature maps are used to encode images [4]. However, this kind of CNN-based preprocessing method requires the deployment of CNN on the sensor, which greatly increases the complexity and cost of the sensor, which is not suitable for VIoT.

In contrast to CS and CNN-based preprocessing, reducing the bit-depth of images can achieve a significant reduction in the amount of visual data with minimal computational overhead. For example, compressing an image with a bit-depth of 16 bits to 4 bits can achieve 75% data compression, and the extra calculation required at the encoder side is only to discard the extra least significant bits. At the decoder side, *Bit-Depth Enhancement* (BDE) technology is used to restore visual signals which have almost the same visual perception quality as the visual perception front end. The asymmetric computing model based on this simple encoding and complex decoding is very suitable for the characteristics of the IoT system which is built from resource-limited PFE and resource-rich computing platforms. It should be noted that compression bit-depth may introduce false contours in the low-bit image. Therefore, false contours must be eliminated as accurately as possible without introducing other artifacts during the reconstruction process.

There are already several BDE algorithms, including context-independent methods, such as *Multiply by Ideal Gain* (MIG) [10] and *Minimum Risk-based Classification* (MRC) [11]. This kind of methods do not make use of context information, so they are not good at suppressing false contours. The context-dependent methods represented by *Contour Region Reconstruction* (CRR) [12] and *Intensity Potential for Image De-quantization* (IPAD) [13] improves the false contour suppression effect by using the information of surrounding pixels, but some non-contour edges are blurred. Methods based on deep learning, such as *Bit-depth Enhancement via Residual Transposed Convolutional Neural Network* (BE-RTCNN) [14,15] and *Deep Reconstruction of Least Significant Bits* (BDEN) [16], use the powerful non-linear fitting capabilities of deep learning to push the performance of the BDE algorithm to a new height. However, more effective network structures, loss functions and training strategies are still worth exploring.

In this paper, we propose a deep convolution network-based BDE method BE-AUTO. It has an auto-encoder structure, where the encoder and decoder are implemented by 5 convolution and deconvolution layers, respectively. This implementation of convolution-combined-with-deconvolution is better than pure convolution or pure deconvolution. In order to achieve better data flow and mitigate the problem of gradient vanishing in deep networks, we add skip connections between the corresponding layers of the encoder and decoder. By using the visual loss proposed in [17] (hereinafter, we collectively call it vgg_loss), BE-AUTO can better suppress false contours than using Mean Square Loss. We find color distortion in some of the result images and that this distortion is caused by out-of-bounds values. To solve this distortion, we add a global skip from the input layer to the output in the network structure. The introduction of the global skip greatly reduces the estimation error, thereby effectively solving the out-of-bounds problem and color distortion. Thanks to BE-AUTO’s convolution-combined-with-deconvolution auto-encoder structure, global skip and vgg_loss, our algorithm can better suppress false contours and color distortion compared with previous algorithms thus achieving the best objective metrics and subjective visual quality.

The main contributions of this article are as follows:

- An auto-encoder of convolution-combined-with-deconvolution structure is proposed for BDE, and it is superior to pure convolution and pure deconvolution. With the help of the global skip connection, the color distortion is well suppressed.
- The mechanism of color distortion is analyzed in detail. It has been experimentally proved that the value of the restored image can be confined into a reasonable range by applying a global skip connection to suppress the color distortion. Moreover, it has been also proved by experimental results that global skip is effective under different network structures.
- A deep BDE algorithm BE-AUTO is proposed. It can effectively suppress false contours and color distortion under the constraint of vgg_loss, and obtain state-of-the-art experimental results both in objective and subjective performance.

The remainder of this paper is organized as follows. Section 2 gives related works and presents our motivation for this work. Section 3 describes our proposed bit-depth enhancement method. The experimental results are presented in Section 4, with ablation analysis in Section 4.3. Section 5 concludes this paper.

## 2. Related Work

BDE is designed to eliminate as many false contours as possible in low-bit-depth input images when reconstructing bit-depth [10,11,18,19,20,21,22,23,24,25,26]. Effective bit-depth enhancement algorithms are essential for energy-efficient VIoT based on bit-depth compression, but the existing BDE algorithms either cannot suppress false contours well or lose much detailed information, so they are not suitable for visual IoT scenarios.

The existing bit-depth enhancement algorithms can be divided into traditional methods and deep-learning-based methods. Traditional methods can be roughly divided into context-independent and context-dependent. Compared with the context-independent methods, the context-dependent methods can better suppress false contours, but have higher complexity. There are few existing BDE algorithms based on deep learning, but they have achieved better results than traditional methods.

Context-independent BDE methods only consider the information of the current pixel when extending the bit-depth. Among them, the easiest way is *Zero-Padding* (ZP). In the ZP algorithm, the *least significant bits* (LSBs) of the input image are directly filled with zeros to achieve the target bit-depth. MIG [10] algorithm multiplies the image value of the low-bit-depth image by an ideal gain: $\left(2^{p}-1\right)/\left(2^{q}-1\right)$ where *p*, *q* are the expected bit-depth and the bit-depth of the input image, respectively. *Bit Replication* (BR) [10] is a simple approximation of the MIG algorithm. It takes the *q* bits of the input image as a whole, periodically attaches it to the latest LSB until the bit-depth is not less than *p*, and then outputs the most significant *p* bits. MRC [11] models bit-depth extension as a minimum classification risk problem. MRC generates an error distribution function of all the possible estimation values and accepts the value that minimizes the associated risk. Context-independent algorithms have low algorithm complexity since they only consider the current pixel. However, due to the lack of use of context information, false contours cannot be effectively suppressed.

Context-dependent BDE methods take into account the context information of the current pixel to effectively suppress false contours. The algorithm *maximum a posteriori estimation of AC signal* (ACDC) [27,28] divides image signal into AC and DC components by constructing graph-Laplace. AC component is estimated based on the *maximum posterior probability* (MAP), which is then used to help the estimation of DC. Because the image’s graph-Laplace takes into account the context information of the pixel, ACDC suppresses false contours better than the context-independent. However, due to complexity considerations, ACDC must divide the image into small patches, which makes it can only use the context information of the current image patch, so the suppression effect is not good. CRR [12] states that if the value of at least one pixel in the four neighborhoods of a pixel is less/greater than that pixel, the pixel is called the upper/lower boundary pixel. The set of adjacent upper/lower boundary pixels is called the upper/lower boundary. CRR finds out all upper/lower boundaries and then estimates the pixel value of the current location based on step_ratio defined by the distances from the current pixel to its closest upper boundary and lower boundary. CRR algorithm could suppress false contours better than previous algorithms. However, in the local maximum/minimum regions, the upper or lower boundary does not exist. Therefore, the false contour suppression effect of these regions is bad. To solve this issue, *Content-Adaptive Image Bit-depth Expansion* (CA) [29] introduces virtual skeletons in these areas to build virtual boundaries. Moreover, CA defines a different step_ratio according to different pixel categories (such as local maximum area pixels, absolute dark pixels), and implements content-adaptive at some level. Regardless of CRR or CA, for a pixel, only the closest upper boundary and lower boundary are active. However, all the boundaries of the flat area where the pixel is located should work for the estimation of the pixel value. Based on this assumption, IPAD [13] makes all the boundaries of the flat region act on the estimation of the current pixel by using the additivity principle of potential, and thus achieving better results. CRR, CA, IPAD improve the suppression of false contours by using context information. However, by converting the estimation of the pixel value of the 2-D image into a 1-D interpolation, the context information of the 2-D image cannot be fully exploited and used. Moreover, CRR and CA update the distance by flooding, making them extremely time-consuming.

Compared with the wide application of deep learning in other image processing fields (such as image super-resolution [30,31,32,33,34]), there are few studies on BDE algorithms based on deep learning. As far as we know, there are only three deep image BDE algorithms: BE-RTCNN [14,15], BDEN [16] and *Concatenating All Level Features* (BE-CALF) [35]. Among them, BE-RTCNN used a full deconvolution residual network and visual loss (loss function based on the visual-geometry-group network (VGG) [36]) to achieve the suppression of false contours. BDEN was a two-column residual network that was used to process flat and non-flat areas in an image, respectively. Because the magnitude of the residuals to be learned was very small, BDEN scaled up the outputs of the two branches separately. Compared with traditional methods, BE-RTCNN and BDEN had achieved better objective indicators and subjective quality. However, the presence of false contours and color distortion could still be seen in the resulting image.

Our method is a little similar to BE-CALF, both using auto-encoder-like network structure and perceptual loss. The difference is that BE-CALF used dense skip connections, but in our method, more efficient symmetric skip connections are used. Another obvious difference is that BE-CALF did not pay attention to the problem of color distortion.

This article is an extension of our previous conference paper [37]. The contents of the extension mainly include the following aspects:

- We make an in-depth analysis of the causes of color distortion, and based on numerical interval estimation and estimation error analysis, theoretical support is provided for the final solution;
- Experiments are made to verify that the global skip connection has similar color distortion suppression effect for different network structures;
- The test set is extended. We add 3 test sets to make a sufficient comparison of different scale BDE in natural and synthetic images between our algorithm and the related;
- The latest related algorithms and 4 visual comparison figures are added to fully reflect the merits of our algorithm.

## 3. Materials and Methods

### 3.1. Problem Model

#### 3.1.1. Quantization and Bit-Depth Variation

The quantizer is an important part of the conversion of image signals from analog to digital. It quantifies the continuous intensity of the light into discrete values, which are then represented by a finite number of bits. The number of bits used per pixel is called the bit-depth of the image, and a common bit-depth is 8 bits. The deeper the bit-depth is, the more realistic the visual quality can be obtained, and of course, the more transmission and storage resources are required.

Quantization methods can be divided into linear quantization and non-linear quantization. Linear quantization is the simplest and most commonly used quantization method. To reduce the number of bits per pixel of an image, a high-bit image needs to be converted into a low-bit image. The conversion process is essentially a re-quantization of the image data. When a high-bit (*p*) image is quantized to a low-bit (*q*) image with the use of linear quantization, a low-bit image can be obtained by discarding *g* LSBs of the high-bit image, where (g=p−q) represents the bit-depth difference. BDE is the reverse of this quantization process. Therefore, BDE is also called image reverse quantization [13].

#### 3.1.2. Key Problems to Be Solved

**Figure 1:**
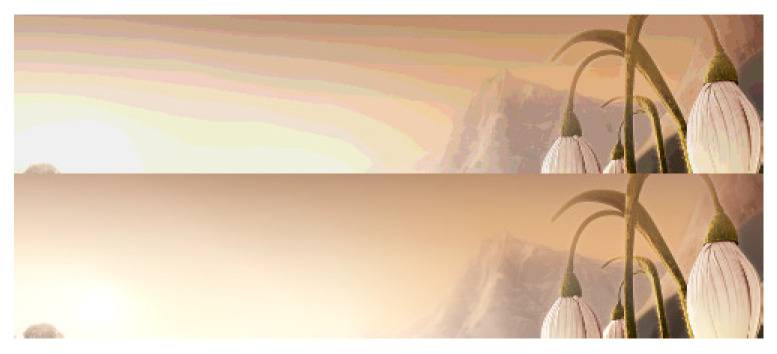
False contour example [37]. The upper part is the reconstructed 16-bit image from 4-bit via ZP and the lower part is the 16-bit ground-truth.

Under the condition of limited bit-depth, if the values of adjacent pixels in the smooth area of the image fall into different quantization intervals, after quantization, an incorrect contour will appear between adjacent pixels. The smaller the quantized bit-depth, the more obvious the false contour. As shown in Figure 1, the lower half of the figure is a high-bit image with a bit-depth of 16 bits, and the upper half is the 4-bit version. As we can see, obvious false contours appear in the low bit-depth image. False contours will severely degrade the visual quality of the image. Therefore, BDE should not only extend the bit-depth but also suppresses false contours, so that the restored image has better visual quality and is as similar to the original high-bit image as possible.

Apart from false contour artifacts, color distortion (as shown by Figure 8c) is another problem to be solved. The direct cause of color distortion is that the estimated values of some components in RGB deviate significantly from the true values. In regression-based BDE methods, such as CNN-based ones, this deviation is more likely to occur, because the optimization target is to minimize the average estimation error.

#### 3.1.3. Mathematical Modeling

As shown in Equation (1), the core purpose of BDE is to find a non-linear mapping function *f* that converts a low-bit input image into a high-bit one. Moreover, during the conversion process, the similarity between the output image and the original high-bit image is maximized with the help of image prior knowledge. In Equation (1), $I^{H/LBD}$ is HBD/LBD image, *s* is similarity measure function, and pr represents image prior. Commonly used image priors include sparsity prior [25], smoothness prior such as ACDC [28] and image content context prior such as CRR [12], CA [29], IPAD [13].

(1) $$\underset{f}{\arg\max}\;s\left(I^{\mathrm{HBD}},\;f\left(I^{\mathrm{LBD}}\right)\right)\;s.t.\;pr\left(I^{\mathrm{LBD}}\right)$$

Deep learning is a data-driven approach. With the help of the loss function and gradient descent, the deep learning network gradually learns the prior information described by the training sample set, and stores it in the form of parameters of the deep network. Therefore, when using the deep network model, the BDE process described in Equation (1) will be rewritten as follows:

(2) $$\underset{\theta }{\arg\min}\;loss\left(I^{\mathrm{HBD}},\;CNN_{\theta }\left(I^{\mathrm{LBD}}\right)\right)$$

where θ represents the network parameters. It should be noted that the prior term in Equation (1) is embodied in the specific form of the loss function in Equation (2). At the same time, the convolutional neural network itself is powerful prior [38].

### 3.2. Loss Function

Loss function is an important part of the neural network. It directly affects the search direction of the network, which decides the network convergence speed and the final performance [39]. Image super-resolution is a field similar to BDE. In image super-resolution, the commonly used loss function is the Mean Square Error loss (mse_loss) [30,32], which is the mean square error of the output image and the label image. mse_loss is widely used in super-resolution, and it has achieved good results. However, false contours cannot be suppressed well [14] in BDE with mse_loss (defined by Equation (3), where $W,H,I,\hat{I}$ are width, height, ground-truth and reconstruction result of an image respectively). In SRGAN [17], the loss function is defined on the feature maps of the output image and the label image in a pre-trained VGG network [36], which allows the resulting image to have more natural texture details. Inspired by this, we define a loss function vgg_loss (defined by Equation (4), where G,I,J are the pre-trained VGG, index of feature map and channel of VGG respectively) on the VGG feature map as the optimization goal of BE-AUTO. It should be noted that vgg_loss is also applied in BE-RTCNN, where it is called visual loss.

(3) $$mse\_loss=\frac{1}{WH}\sum_{x=1}^{W}\sum_{y=1}^{H}{\left(I_{x,y}-{\hat{I}}_{x,y}\right)}^{2}$$

(4) $$vgg\_loss=\sum_{i=1}^{I}\sum_{j=1}^{J_{i}}\sum_{x=1}^{W_{i,j}}\sum_{y=1}^{H_{i,j}}\frac{1}{IJ_{i}W_{i,j}H_{i,j}}{\left(G{\left(I\right)}_{x,y}^{i,j}-G{\left(\hat{I}\right)}_{x,y}^{i,j}\right)}^{2}$$

### 3.3. BDE Model

To obtain results of excellent subjective and objective quality, the deep BDE algorithm must suppress the false contour and overcome the color distortion at the same time.

**Figure 2:**
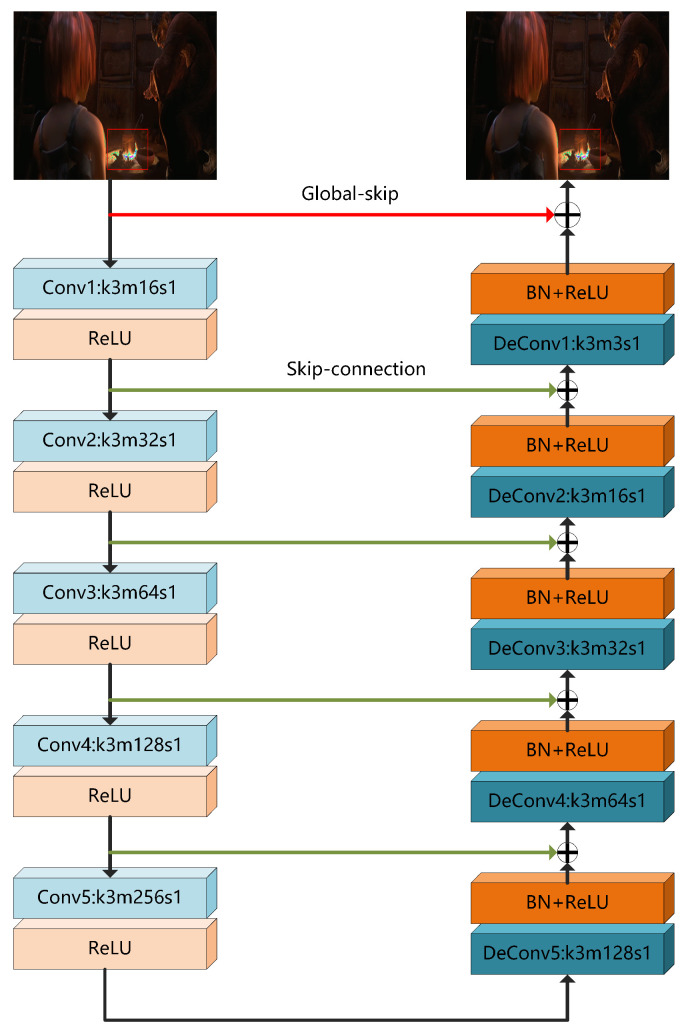
The network structure of the proposed algorithm BE-AUTO [37].

The proposed scheme is presented in Figure 2. As shown in Figure 2, BE-AUTO is a convolution neural network-based bit-depth enhancement method with an auto-encoder-like structure. It takes RGB images as input and output RGB ones. It consists of an encoding module (left part) and a decoding module (right part). The encoding module is implemented by 5 (can be increased or decreased as needed) convolution layers. The size of convolution kernels (k∗) are all 3 × 3, and from the first layer to the fifth layer, the number of convolution kernels (m∗) is doubled every layer from 16 to 256 to extract richer features. The decoding module is implemented by 5 deconvolution layers [40], and the size of the deconvolution kernel is also 3 × 3. The number of convolution kernels is 128, 64, 32, 16, 3 from the fifth layer of the deconvolution to the first. Details will lose if the sizes of feature maps decrease, so we set stride (s∗) as 1. To alleviate the disappearance of the gradient and facilitate the training of the deep network, we add a *Batch Normalization* (BN) layer [41] behind each deconvolution layer and add a skip connection [42] between the corresponding layers of the codec module. The activation functions used in BE-AUTO are ReLU [43]. The reason the convolution and deconvolution are used to implement the codec module is that the convolution and deconvolution functions fit well with the codec, which will be analyzed and proved in Section 4.3.5. Based on the analysis of color distortion, we short connect the input to the output of the decoding module, and element-wisely add them up to get the final output. To distinguish it from the connection mentioned earlier, we call this connection “global skip”. Global skip will play a key role in suppression color distortion as shown in Section 4.3.4, because it can significantly reduce estimation errors (see Section 4.3.1 for more details).

## 4. Experiments and Discussion

### 4.1. Experiment Settings

#### 4.1.1. Data Sets

The test sets include sintel-8, fiveK-40, BDE-set, Kodak dataset [44]. Sintel-8 contains eight 16-bit images randomly selected from sintel [45] for testing large-scale artificial image BDE from 4-bit to 16-bit. The fiveK-40 consists of forty 16-bit natural images randomly selected from fiveK [46] for testing 4-bit to 16-bit enhancement of natural images. BDE-set and Kodak datasets contain eight and twenty-four 8-bit images respectively for testing small-scale BDE from 6-bit to 8-bit. BDE-set is a commonly used dataset in traditional BDE (like [11]). Since its quantity is small, BDE-set is supplemented with Kodak dataset.

Two training sets sintel-2000 and DIV2K [47] are used to train the network for 4-bit to 16-bit and 6-bit to 8-bit BDE, respectively. The sintel-2000 has 2000 frames selected from [45] and each frame has a resolution of 436 × 1024 and a bit-depth of 16. Sintel-2000 has no intersection with sintel-8. The DIV2K contains eight hundred high-quality 8-bit natural images with a resolution of 1404 × 2040.

#### 4.1.2. Algorithms to be Compared

The proposed algorithm is compared with the representative BDE methods, including: zero-padding (ZP), MIG [10], BR [10], MRC [11], ACDC [27,28], CRR [12], CA [29], IPAD [13], BE-RTCNN [14,15] and BDEN [16]. Of these methods, only BE-RTCNN and BDEN are deep learning-based methods, and the rest are traditional methods. It should be noted that the experimental data of BDEN and IPAD are the results of running the code provided by the papers’ authors.

#### 4.1.3. Training Details

The network training process is performed on an NVIDIA 1050Ti. A 96 × 96 patch is randomly cropped from each image. These patches form batches of size 8, and are fed into the network for training. Epoch is set to 120 for a good tradeoff between training time and performance. The learning rate is set to 1 × 10${}^{-4}$. As for optimizer, we choose Adam with beta1=0.9. Each 16/8-bit image is linearly quantified into a 4/6-bit image. Then, the 4/6-bit image is restored back to a 16/8-bit image through the Zero-Padding method, then fed into the network for training.

### 4.2. Results and Discussion

#### 4.2.1. Objective Performance Evaluation

**Figure 3:**
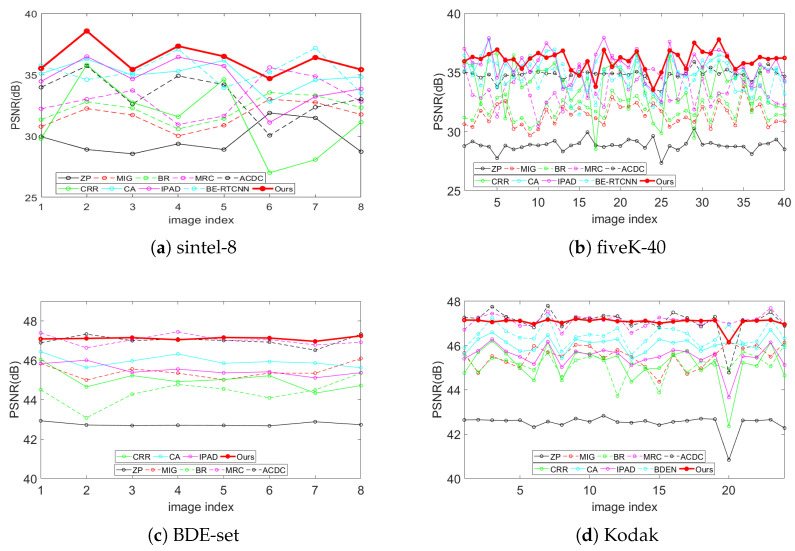
PSNR curve s comparison with state-of-the-art on four test dataset.

Table 1 lists the PSNRs (computed on RGB color space) for all methods in comparison on the test sets sintel-8, fiveK-40, BDE-set and Kodak. The best result is highlighted in bold and the second best is underlined. A comparison of the PSNR curves on test datasets is shown in Figure 3.

It can be seen from Table 1 and Figure 3 that our method achieves superiority to other algorithms in large-scale BDE scenes from 4-bit to 16-bit for both natural images (fiveK-40) and artificial images (sintel-8). In small-scale scenarios from 6-bit to 8-bit, MRC and ACDC show significant advantages over other traditional methods, especially on the Kodak test set. This is most likely because that in this scenario, there are only four possible values for the newly added two bits, which greatly reduces the risk of classification error of MRC and the error probability of the AC component of ACDC. This is just the reason these two algorithms do not perform well in large-scale scenarios. For small-scale scenarios, our algorithm has achieved the best results on both BDE-set and Kodak, with a gain of 0.15 dB compared with the second-best algorithm. In summary, our algorithm achieves better objective results than these comparison algorithms for all scales and image types. This is due to the following three aspects:

(1) The use of convolution for encoding and deconvolution for decoding can better extract features and perform better reconstruction.
(2) The addition of the global skip connection allows the network output to remain well within the desired range, thereby avoiding color distortion and not interfering with the false contour suppression.
(3) vgg_loss has an incomparable inhibitory effect on false contours relative to mse_loss, which is fully discussed in BE-RTCNN [15].

#### 4.2.2. Subjective Performance Evaluation

**Figure 4:**
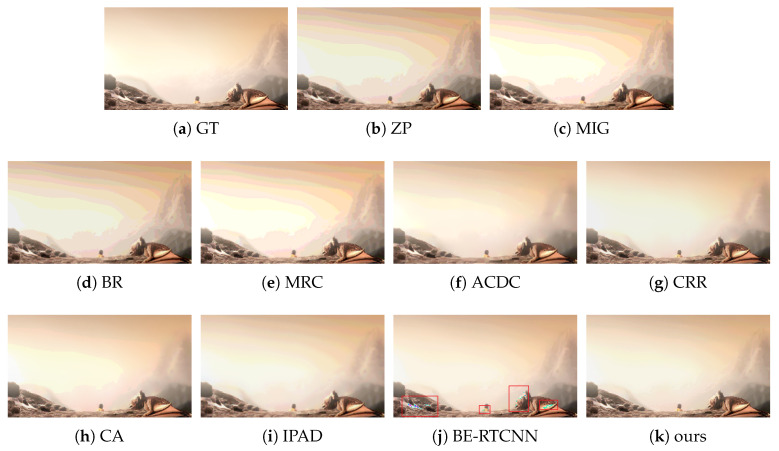
The BDE results on 4bit-16bit synthetic image from sintel-8 dataset [37].

**Figure 5:**
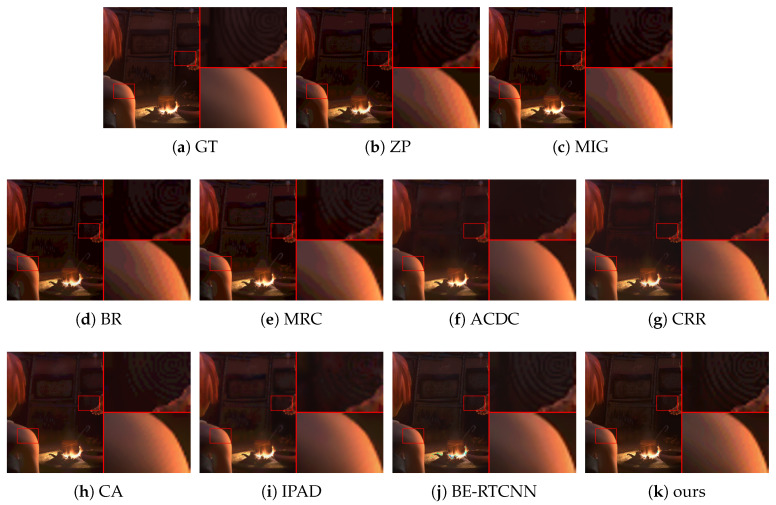
The BDE results on 4bit-16bit synthetic image from sintel-8 dataset.

**Figure 6:**
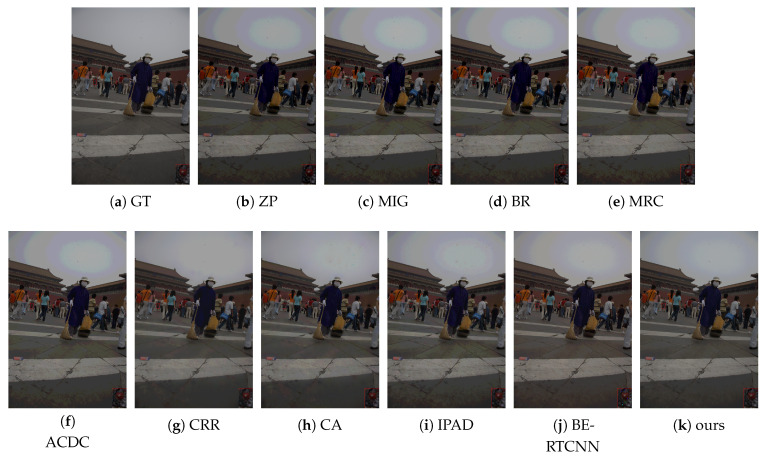
The BDE results on 4bit-16bit natural image from fiveK- dataset.

**Figure 7:**
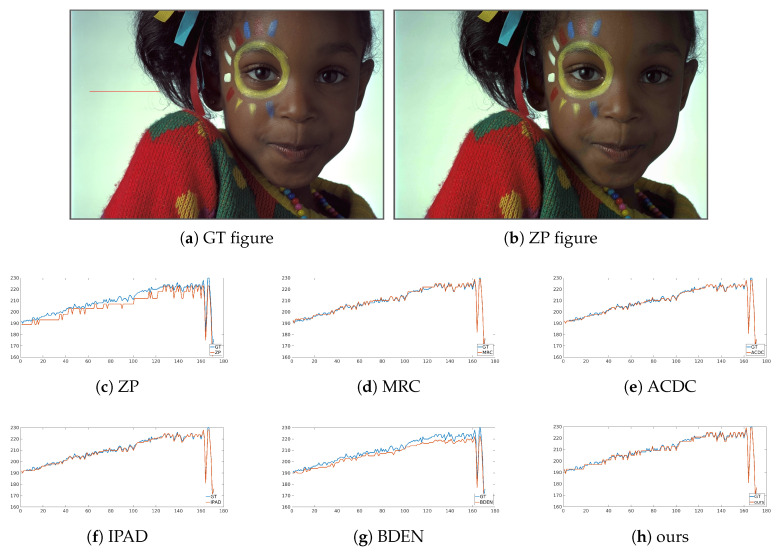
Intensity curves of 6bit-8bit natural BDE image from Kodak.

Subjective performance comparisons with state-of-the-art are depicted in Figure 4, Figure 5, Figure 6 and Figure 7. Where Figure 4 and Figure 5 show the comparison of artificial image BDE on a large scale (from 4-bit to 16-bit). Large-scale natural image BDE comparison from 4-bit to 16-bit is depicted in Figure 6. For 6-bit to 8-bit small-scale BDE (Figure 7), the difference between the 6-bit image and the 8-bit ground-truth is already hard to distinguish (as depicted by Figure 7a,b) by human eyes, so we use pixel value curves to show the reconstruction’s similarity with the ground-truth [16].

It can be seen from Figure 4 that the results of the traditional methods have different degrees of false contours. Although CRR suppresses false contours well, the results are over-smoothed (such as the hilltop). Compared with the traditional method, under BE-RTCNN, false contours can be suppressed well, but significant color distortion occurs. That is because of the lack of global skip connection to effectively limit the range of values, resulting in numerical flipping.

In Figure 5, to better show the suppression effect of false contours and the degree of detail preservation by different methods, we enlarge one smooth area and detail area circled by the red boxes 4 times and paste them on the right side of the image. It can see from Figure 5b–e that ZP, MIG, BR and MRC perform poorly in suppressing false contours in smooth regions, but they can maintain the real contours of detail regions well. ACDC, CRR and IPAD are just the opposite. CA’s detail maintenance is better than that of CRR, but it is still not satisfactory. BE-RTCNN does a good job of suppressing false contours and maintaining details, but it has obvious color distortion. Our method achieves the best tradeoff between false contour suppression, color distortion suppression, and detail preservation, thus producing the best visual quality.

Our algorithm has obvious advantages in false contour suppression and avoiding color distortion. The superiority of our method can also be seen from Figure 6. Compared with the original image, except the method proposed in this paper, the bricks and zebra lines in the recovered image of all other algorithms have different degrees of tonal distortion. Color distortion occurred in the resulting image of BE-RTCNN. For better viewing, we randomly select one distortion region, zoom in it by 4 times, and stick it at the bottom right of the image with a red box to highlight it.

Figure 7a is one image fetched randomly from Kodak data set. Figure 7b is the restored images by ZP. To further reveal the suppression effect of false contours, a row of data extracted from the smooth area of the recovered image and the original data of its corresponding position are plotted in Figure 7c–h for comparison. Obvious steps appear in the ZP’s curve as shown by Figure 7c. Those steps indicate false contours. Steps are well smoothed in Figure 7d–f. This is consistent with the high PSNR listed in Table 1. BDEN’s curve (Figure 7g) has a similar shape with ground-truth but has obvious value offset from left to right. This may be because flat and non-flat areas are reconstructed separately, in order to make a smooth transition between flat and non-flat areas, and make the boundary of the regions more natural, so the fusion step made some kind of value convergence. As shown in Figure 7h, the result obtained by our method maintains good consistency with the ground-truth data in both the shape and numerical value.

As shown in Table 1 and Figure 4, Figure 5, Figure 6 and Figure 7, our method can suppress false contours and color distortion well, and preserve details at the same time, thus having state-of-the-art objective metric PSNRs and subjective visual perception quality. This comes from the encoder-decoder network model implemented by convolution and deconvolution, global skip connection and the use of vgg_loss.

#### 4.2.3. Run Time Comparison

To measure the computational complexity, we test the run times of the algorithms in comparison on the testset sintel-8 with resolution 500 × 218. We test on a platform with Intel Core${}^{\mathrm{TM}}$ i5-7500 CPU @ 3.40 GHz × 4 and Samsung DDR4 8 GB RAM @ 2666 MHz. Run times are listed in Table 2. As can be seen from Table 2 that our algorithm has a significant run time advantage over both traditional algorithms and related deep learning algorithms.

### 4.3. Ablation Analysis

#### 4.3.1. Analysis of Color Distortion

**Figure 8:**
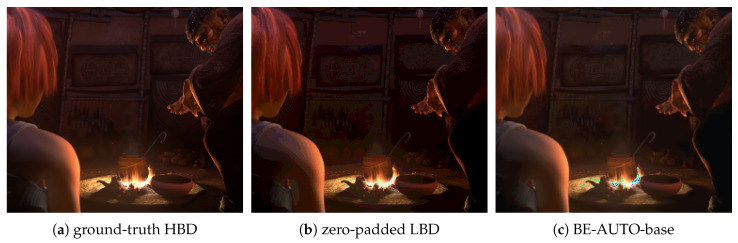
False contour suppression and color distortion [37]. (**a**) is the ground-truth 16-bit image; (**b**) is the zero-padded fake 16-bit image from 4-bit; and (**c**) is the result of BE-AUTO-base.

As we can see from these three sub-figures (j) of Figure 4, Figure 5 and Figure 6, the results of BE-RTCNN [14] have obvious color distortion. This type of color distortion is not unique for BE-RTCNN, but shows some kind of universality for CNN-based BDE algorithms. To show this, we delete the global skip connection of BE-AUTO while keeping all the other components the same to get a new solution, which we call BE-AUTO-base. Figure 8c depicts the result of BE-AUTO-base. The same kind of color distortion as that of BE-RTCNN occurs as we compare it (Figure 8c) with the ground-truth (Figure 8a) and Figure 5j.

Visual quality would severely degrade if there is color distortion. BDE’s application scenarios, such as high-bit display, are hope ed to present better visual quality, which means that the visual quality of the resulting image is an important metric for evaluating BDE algorithms. Therefore, to achieve better visual quality, both color distortion and false contours should be effectively suppressed.

In this section, we will analyze the causes of color distortion and the way to suppress color distortion without affecting the suppression of false contours. We will also show that the traditional truncation method cannot effectively solve this problem.

#### 4.3.2. Color Distortion Reasons

Just as with other deep learning (such as EDSR [33]) algorithms for image processing, we preprocess the data range before training and testing to avoid interference with the network by the numerical range of the input images. In this article, we will map the pixel values of the input and label images to [−1,1] according to Equation (5). Therefore, it is reasonable to expect that the value range of the network’s output is also [−1,1]. Moreover, because the data type is unsigned integer when the image is stored and displayed, the output of the network is processed by Equations (6) and (7) and then stored, displayed, and to compute objective metrics (such as PSNR).

(5) $$map\left(X\right)=X/max\_value-1\;where\;max\_value=2^{p}-1$$

(6) remap(X)=(X+1)/2×max_value

(7) X=uint(X)

However, when we check the output of the BE-AUTO-base, we find that the value range of the output image exceeds the expected interval [−1, 1]. The numerical ranges of the eight output images are shown in Table 3. Once the value crosses the boundary and is processed by Equation (6), a value greater than max_value or smaller than 0 will appear. As is shown in Equation (8), after these out-of-bounds values are type-converted, numerical flip occurs, and the image’s color will distort. In addition, if the estimated value of any color component significantly exceeds the interval determined by the true value, the ratio of the three color channels will be out of balance and results in color distortion.

(8) $$uint16\left(x\right)=\left\{\begin{array}{c} \left\lfloor x\rfloor \;\;\;\;\;x\in [0,65,535\right]\\ \left\lfloor x-65535\rfloor \;x\in (65,535,+\infty \right]\\ \left\lfloor x+65536\rfloor \;x\in (-\infty ,0\right) \end{array}\right.$$

#### 4.3.3. Non-Triviality of Color Distortion Problem

**Figure 9:**
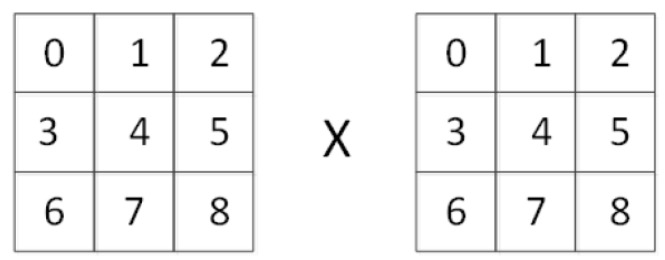
Example of image patch and convolution kernel. The number in each box is the index of corresponding pixel or the convolution kernel’s weight.

Convolution neural network is a process of sliding convolution. To explore the underlying reasons for the values exceeding the range, we take a convolution operation as an example. Also, for ease of analysis, the bias and activation of the convolution neural network are ignored. As is shown in Figure 9, the left part is the image block to be convolved and the right part is a 3 × 3 convolution kernel. Therefore, the convolution operation can be expressed by Equation (9):

(9) $$y=\sum_{i=0}^{8}\omega_{i}\times x_{i}$$

where *y* is the output value, $x_{i}$ is the input pixel, $w_{i}$ is the weight, and the index number *i* is as shown in Figure 9. The ground-truth value of *y* called $\hat{y}$ lies in $\left[x_{4},x_{4}+b\_g-1\right]$, where $b\_g=2^{g}$ is the quantization step, g=p−q is the difference between the target bit-depth *p* and the source bit-depth *q*. Therefore, the training process of the convolution neural network essentially seeks a set of parameters *W* to map each group of *X* to a variation interval $\left[x_{4},x_{4}+b\_g-1\right]$. Since $x_{i}$ belongs to [0,b_g∗max_q], where $max\_q=2^{q}-1$ is the maximum pixel value of the source image, and the target range *T* for a given pixel $x_{4}$ is

(10) $$T=[x_{4},x_{4}+b\_g-1]$$

then the target range for the whole image UT is the union of all *T*, i.e.,

(11) $$UT=\cup_{x\in [0,b\_g\times max\_q]}T=\left[0,b_{g}\times max\_q+b\_g-1\right]=\left[0,2^{p}-1\right]$$

Taking 4-bit to 16-bit BDE as an example, $UT=\left[0,2^{16}-1\right]=\left[0,65,535\right]$, which means that the neural network needs to have the ability to map the input image to [0,65,535], although the value of each output pixel is theoretically only within *T* = $\left[x_{4},x_{4}+b\_12-1\right]=\left[x_{4},x_{4}+4095\right]$. *T* and UT have a huge difference in the length of the interval. Moreover, the convolution neural network not only needs to cope with the prediction of a certain pixel but with different images and different pixels. Therefore, to minimize the average prediction error over the entire image and throughout the data set for better generalization ability, the training process must focus on UT and ignore *T*. So, when *T* and UT are different, it is bound to sacrifice prediction accuracy for a certain pixel. This kind of sacrifice is reflected as that the prediction result is not within the interval $\left[x_{4},x_{4}+b\_g-1\right]$ in the prediction of a certain pixel, and is not in the interval $\left[0,2^{p}-1\right]$ when predicting for multiple pixels or the whole picture. This out-of-bounds eventually manifests itself as color distortion. Therefore, to suppress color distortion, the difference between *T* and UT should be minimized and be identical in the ideal situation. From Equations (10) and (11), we can see that when *T* is independent of $x_{4}$, UT=T.

The color distortion problem can also be analyzed from the perspective of estimation error. We decompose a *p*-bit prediction of the value *y* into the most significant *q*-bits and residual *g*-bits, called *h*, *r*. therefore:

(12) y=b_g×h+r

Among them, *h* lies in $\left[0,2^{q}-1\right]$, and *r* lies in $\left[0,2^{g}-1\right]$. Performing error analysis on *y*, we can get:

(13) Δy=b_g×Δh+Δr

When p=16,q=4, Equation (13) is instantiated as Δy=4096×Δh+Δr. From Equation (13), we can see that a very small error in *h* can make a large error in the estimation of *y* because Δh is significantly magnified by b_g. Therefore, to effectively suppress color distortion, it is necessary to suppress the estimation error of the most significant 4 bits. If an accurate estimate of the 4 bits is obtained, the numerical out-of-bounds and color distortion can be effectively suppressed. We can see that the correct estimate of the high 4 bits *h* is equal to the high 4 bits of $x_{4}$, because $x_{4}$ is obtained by bit-shifting the 16 bits $\hat{y}$ by 12 bits, the high 4 bits of $x_{4}$ and $\hat{y}$ are identical). Therefore, when estimating *y*, if we can get $x_{4}$, we only need to estimate the lower 12-bits, and we can make *T* independent of $x_{4}$.

In previous CNN-based algorithms, the network output is truncated. That is, the value not within the interval is forcibly assigned to the nearest interval boundary value. This operation is based on the prior that the value represented by finite bits is limited. Taking *p*-bit image as an example, the range of pixel values that can be represented is $\left[0,2^{p}-1\right]$, so predicted values greater than $2^{p}-1$ or less than 0 are false predictions. Re-assigning these out-of-bounds values to the nearest interval boundary not only enables the predicted value to be represented by the finite bits but also reduces the estimation error. However, with the analysis of the out-of-bounds causes in the above two paragraphs, we can find that the numerical truncation can only deal with the out-of-bounds for the full interval $\left[0,2^{p}-1\right]$, but cannot deal with the out-of-bounds for $\left[x_{4},x_{4}+b\_g-1\right]$ that occurs when predicting certain pixel. Therefore, numerical truncation can only be used as an auxiliary solution. To better resolve numerical out-of-bounds and color distortion, we should minimize the estimated error of *q*-bits or make *T* unrelated to $x_{4}$, and in this paper, we propose to use global skip to achieve this.

#### 4.3.4. Effect of Global Skip Connections

**Figure 10:**
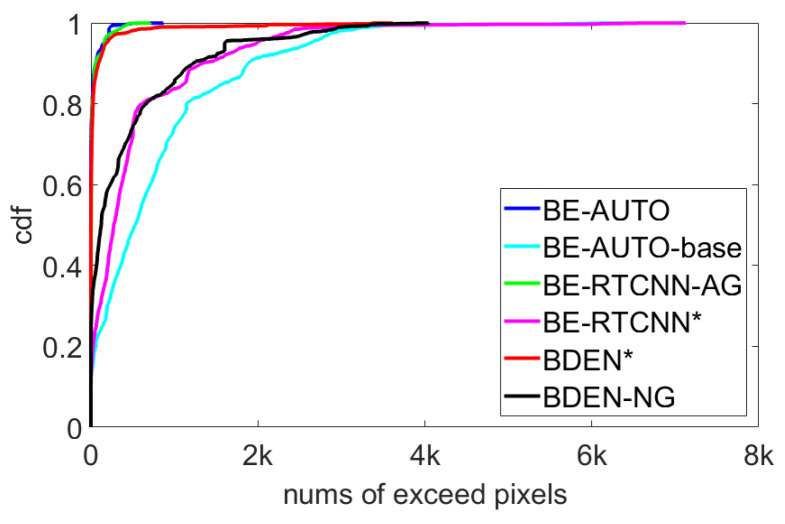
CDF of the number of out-of-bounds pixels for three network structures with and without global skip connections. * refers to our implemented version.

The difference between BE-AUTO and BE-AUTO-base is that the former has a global skip connection. This is a very simple operation, but it is very effective. Moreover, the global skip connection is universal for the suppression of color distortion and can stably improve network performance. Global skip connections have also been used in other network structures, such as VDSR [32] in the image super-resolution domain, and the recent deep BDE algorithm BDEN [16], but neither functionality nor universality of global skip connections are proven. We will compare and verify on the existing three deep BDE networks (BE-AUTO, BE-RTCNN, BDEN). Among them, BE-RTCNN has no global skip connection, and BDEN has. Because BE-RTCNN does not provide code, and BDEN’s code is an encrypted file, we implemented it as described by the papers. In Figure 10, BE-RTCNN* and BDEN* refer to our implemented version, and BE-RTCNN-AG refers to the addition of a global skip connection. BDEN-DG refers to the deletion of the global skip connection. Figure 10 is the *Cumulative Distribution Function* (CDF) of the number of out-of-bounds pixels for three network structures with and without global skip connection on 1909 randomly selected images of size 436 × 1024. The average number of out-of-bounds pixels are listed in Table 4. It can be seen from Figure 10 that for each network structure, the output value range of scheme without the global skip connection has a significant out-of-bounds, and after the global skip connection is added, it can be well maintained within the predetermined interval, avoiding color distortion. It can be seen that the global skip connection has a significant effect on the definition of the output value range and the avoidance of color distortion. This effect is independent of the network structure and is universal. Therefore, when performing a deep BDE network design, the global skip connection can be used as a regular component. The experimental results here are consistent with the analysis in Section 4.3.1.

#### 4.3.5. Convolution vs. Deconvolution

**Figure 11:**
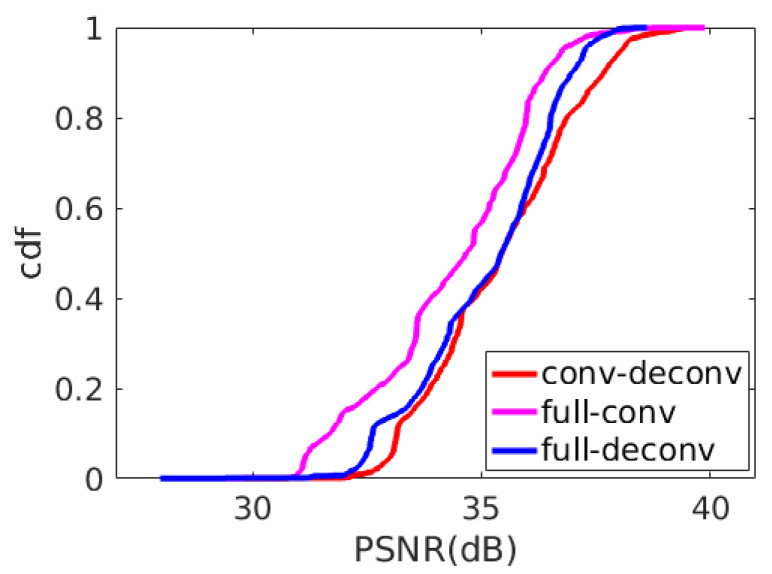
CDF of PSNR for network structure implemented with convolution or deconvolution on 1886 random selected images.

Convolution and deconvolution are a set of reverse operations. Convolution can be seen as a “bottom-up” process, such as extracting edges, contours and even objects from low-level features to high-level features from the input image. Deconvolution is a “top-down” [40] process. The idea of deconvolution is to backtrack what kind of low-level features can produce current characteristics. The level-by-level abstraction of convolution and step-by-step reconstruction of deconvolution corresponds to the encoding module and decoding module of the auto-encoder well, which is the basic idea of designing BE-AUTO. This idea has also been verified experimentally. In Figure 11, full-conv means that the codec module is implemented with convolution layers. Full-deconv means that the codec is implemented by deconvolution. In addition, conv-deconv refers to encode with convolution and decode with deconvolution layers, which is the scheme adopted by BE-AUTO. These three schemes are consistent except for the convolution or deconvolution layers. Figure 11 shows the CDF of PSNR of these three implementations on 1886 images selected randomly from sintel-2000 and fiveK. The average PSNRs of these three scenarios are listed in Table 5. As we can see from Figure 11, conv-deconv scheme has a significant advantage over the full-conv, because conv-deconv’s CDF curve (the red line) lies at the right side of the full-conv’s (the pink line) with a big gap. The full-deconv scheme (the blue line) has similar performance to that of the conv-deconv scheme at the low PSNR level, but gradually falls behind with the increase of PSNR. This shows that the conv-deconv solution can stably improve the reconstruction quality of the test image compared with the other two schemes, so we chose conv-deconv (i.e., convolution-combined-with-deconvolution) at the final scheme.

## 5. Conclusions

In this paper, an auto-encoder-like CNN model is proposed to suppress color distortion and false contours in images of bit-depth enhancement. Based on this model, a simple bit-depth re-quantization method can be used in PFEs of visual IoT to significantly decrease the visual data volumes with extremely low computation complexity and low energy consumption and achieve high visual perception performance of the restored visual image. The obtained experimental results reveal that our method can obtain better results compared to other competing methods in subjective and objective performance. Future work will focus on integrating the model into HEVC reference frame and reducing the calculation complexity of the model.

## Figures and Tables

**Table 1:** Comparison with state-of-the-art on PSNR.

Dataset	ZP	MIG	BR [10]	MRC [11]	ACDC [28]	CRR [12]	CA [29]	IPAD [13]	BE-RTCNN [15]	BDEN [16]	Ours
sintel-8	29.7141	31.6352	32.1936	33.0923	33.3474	31.3352	34.9920	34.4867	35.2994	-	**36.2270**
fiveK-40	28.8342	31.3927	31.9534	33.6832	34.8084	33.8474	35.2481	35.5903	34.3688	-	**36.0413**
BDE-set	42.7506	45.4445	44.3908	47.0334	47.0099	45.0150	45.9547	45.5052	-	-	**47.1877**
Kodak	42.5077	45.5028	45.1540	47.1177	47.1001	45.1564	46.0462	45.5074	-	46.4550	**47.2576**

**Table 2:** Average run time (in seconds) of different algorithms.

Algorithm	ZP	MIG	BR [10]	MRC [11]	ACDC [28]	CRR [12]	CA [29]	IPAD [13]	BE-RTCNN [15]	BDEN [16]	Ours
Run time	0.0005	0.0012	0.0041	90.4498	332.0405	15.9587	24.476	7.2593	1.9025	2.6334	1.1031

**Table 3:** Output range of BE-AUTO-base on 8 random selected images.

img_idx	img1	img2	img3	img4	img5	img6	img7	img8
range	[−1.16,1.21]	[−0.94,0.76]	[−1.14,1.18]	[−1.18,1.01]	[−1.21,1.05]	[−1.17,1.04]	[−1.18,0.97]	[−1.29,1.18]

**Table 4:** Average numbers of out-of-bounds pixels of three network structures on 1909 randomly selected images with and without global skip connections.

	BDEN	BE-RTCNN	Ours
without global skip	415.62	495.34	761.04
with global skip	53.13	26.36	20.27

**Table 5:** PSNR of conv/deconv on 1886 random selected images.

	Full_Conv	Full_Deconv	Conv_Deconv
average PSNR	34.3741	35.1176	35.4293

## Data Availability

Publicly available datasets were analyzed in this study. This data can be found here: http://r0k.us/graphics/kodak/, https://www.xiph.org/, and at https://data.csail.mit.edu/graphics/fivek/.
